# Scientific Abstracts from the Phoenix Children’s 13th Annual Fetal Cardiology Symposium December 8–11, 2022 at The Phoenician, Scottsdale, AZ

**DOI:** 10.1007/s00246-023-03110-3

**Published:** 2023-03-02


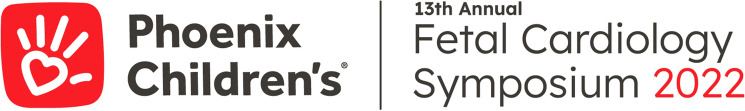


Organizing Committee: Christopher Lindblade, MD, Anita Moon-Grady, MD, Norman Silverman, MD, Julia Solomon, MD

Sponsor Note (disclosure): Publication of this supplement was funded by Phoenix Children’s. All content was reviewed and approved by the 13th Annual Fetal Cardiology Symposium Organizing Committee, which held full responsibility for the abstract selections.

## Treatment, Rather than Delivery, of the Early Term Fetus with Supraventricular Arrhythmia

Samantha Kops, M.D.,^1^ Lisa Hornberger, M.D.,^3^ Edgar Jaeggi, M.D.,^4^ Lisa Howley, M.D.,^5^ Orsolya Gilicze,^6^ Anita Moon-Grady,^6^ Orhan Uzun, M.D.,^7^ Alex Kaizer, Ph.D.,^8^ Bettina F. Cuneo, M.D.^1,2^

^1^The Heart Institute and ^2^Colorado Fetal Care Center, Children’s Hospital Colorado, University of Colorado, Aurora, CO; ^3^Division of Cardiology, Department of Pediatrics Stollery Children’s Hospital, University of Alberta, Alberta, CA; ^4^Division of Cardiology, Department of Pediatrics, Hospital for Sick Kids, University of Toronto, Toronto, CA; ^5^Children’s Hospital Minnesota, Minnesota, MN; ^6^University of California San Francisco, San Francisco, CA; ^7^School of Medicine and the University Hospital of Wales, Cardiff, UK; ^8^Department of Biostatistics and Informatics, University of Colorado, Aurora, CO.

**Objectives**: In many centers, patients with fetal supraventricular arrhythmias (SVA) presenting at 35–39 weeks are delivered by caesarian section, rather than treated in utero to restore sinus rhythm and undergo a vaginal delivery. We reviewed our results of fetuses presenting with SVA at ≥ 35-weeks undergoing treatment. **Methods: **We performed a multicenter retrospective case series between 2015 and 2021 of ≥ 35-week fetuses with SVA treated rather than delivered. We collected presenting and delivering gestational age (GA), diagnosis, treatment, time to normal rhythm, type of delivery and postnatal SVA recurrence. Results were presented as mean ± SD. **Results:** Thirty-seven fetuses presented at 35–39 (36.1 ± 1.1) weeks (table); 4 (11%) were hydropic. In utero treatment was successful in 35 (97%) including 3/4 with hydrops (figure) using flecainide (n = 11), digoxin (n = 7), sotalol (n = 11), digoxin and sotalol (n = 1), flecainide and sotalol (n = 1) or digoxin and flecainide (n = 6). Hydropic fetuses responded in 1–2 days with sotalol (2) or sotalol and digoxin (1); one hydropic fetus remained in atrial flutter. Despite a high rate of conversion, only 23 (62%) patients underwent a vaginal delivery including 15/23 with short RP tachycardia (65%), 3/23 with long RP tachycardia (13%) and 5/23 with atrial flutter (22%). Caesarian section was performed in 14 patients (12 in sinus rhythm): 2 because of resistant SVA, 3 with atrial ectopy or sinus bradycardia, and 9 for obstetrical reasons. Postnatal antiarrhythmic treatment was given in 21 patients, including 3/4 with hydrops that had resolved in utero. **Conclusion:** In utero treatment of the ≥ 35-week fetus with SVA is highly successful even in the hydropic fetus. However, operative delivery in this population rate remains high, and further studies are needed to define the reasons. A randomized trial of delivery vs. treatment would lead to evidence-based guidelines helpful in management.

**Table:** Table: Outcomes of prenatal antiarrhythmic treatment of the ≥ 35-week fetus with supraventricular arrhythmia (SVA).

DX	Gestational Age at Presentation (wks)	Time to Convert (days)	Gestational Age at Delivery (wks)	NSVD (n = 23)	CS OB Reasons (n = 9)	CS Arrhythmia (n = 5)	Postnatal SVA (57%)
Short RP (n = 20)	36.1 ± 0.95	3.85 ± 3.92	38.4 ± 1.14	15/20 75%	3/9 33%	2/5 40%	13/20 65%
Long RP (n = 7)	36.2 ± 1.61	5.71 ± 5.50	38.5 ± 1.10	3/7 43%	3/9 33%	1/5 20%	5/7 71%
Flutter (n = 10)	36.2 ± 1.27	3.25 ± 5.57	38.3 ± 0.95	5/10 50%	3/9 33%	2/5 40%	3/10 30%

*IUFD* intrauterine fetal demise, *VIP* voluntary interruption of pregnancy, *PVFR-VTI* pulmonary vein forward/reverse velocity time integral

**Figure:**
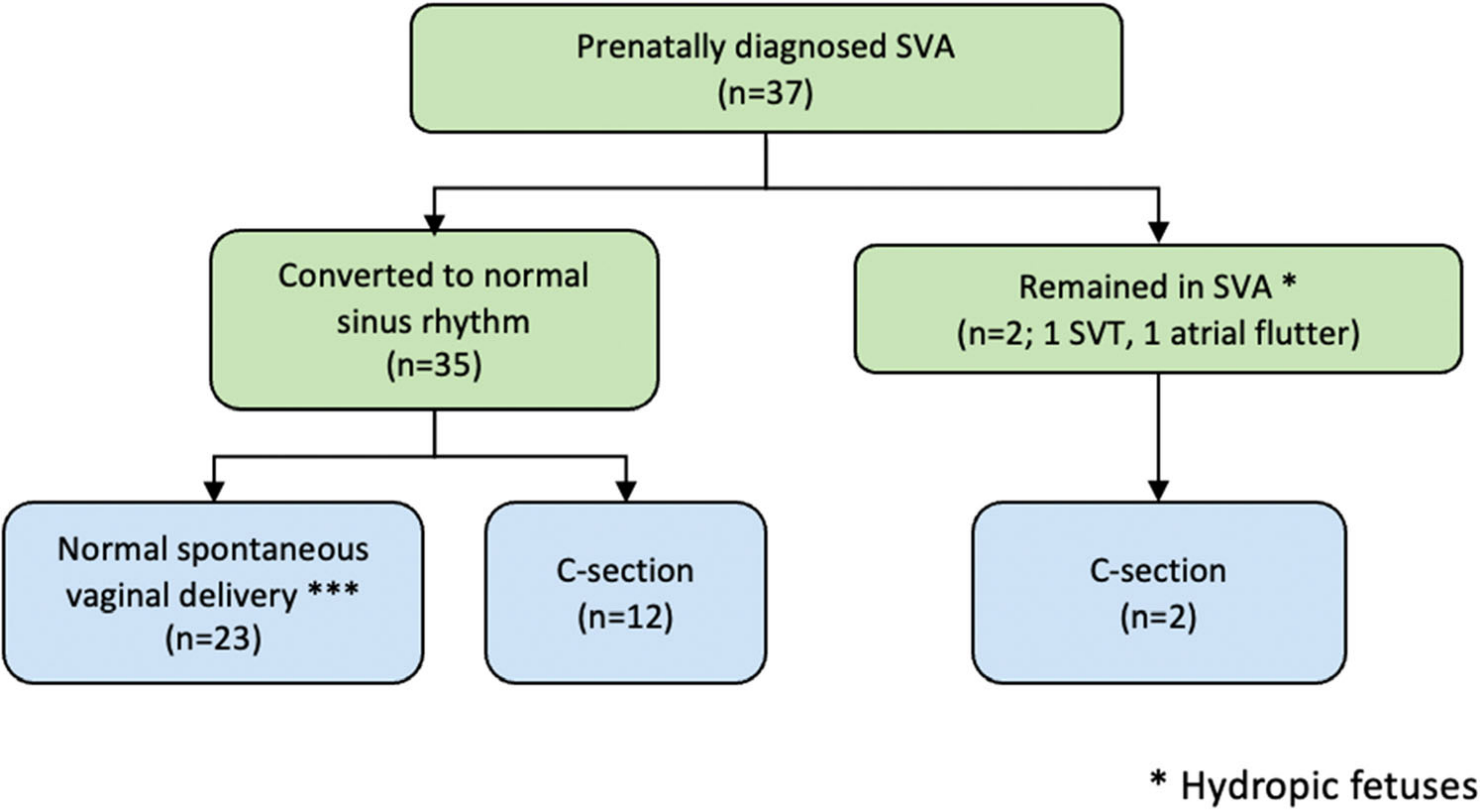
Delivery outcomes of prenatal antiarrhythmic treatment of the ≥ 35-week fetus with supraventricular arrhythmia (SVA).

## Trends in Pulmonary Vein Doppler and Size Throughout Gestation in Fetuses with Hypoplastic Left Heart Syndrome

**Presenting/primary author:** Michael Nguyen, DO – Advanced Pediatric Cardiac Imaging Fellow, Children's Hospital of Colorado (Orchid ID 0000–0001-6206–2926).

Coauthor: Samantha Kops, MD – Pediatric Cardiology Fellow, Children's Hospital of Colorado Coauthor: Alexander Kaizer, PhD – Assistant Professor, School of Public Health, University of Colorado. Coauthor: Lisa Gilbert, BS – Children's Hospital of Colorado. Coauthor: Camila Londono-Obregon, MD – Instructor, University of Colorado School of Medicine. Coauthor: Karrie Villavicencio, MD – Associate Professor, University of Colorado School of Medicine. Coauthor: Bettina Cuneo, MD – Professor, University of Colorado School of Medicine. Coauthor: Michelle Grenier, MD – Visiting Professor, Children's Hospital of Colorado School of Medicine.

**Disclosures**: None of the authors have any disclosures competing with interest related to the study.

**Background:** Newborns with hypoplastic left heart syndrome (HLHS) and restrictive/intact atrial septum (RAS) have a high mortality. Pulmonary vein Dopplers showing forward/reverse velocity time integral (PVFR-VTI) < 5 in utero are predictive for the need for emergent atrial septostomy (EAS), but little is known about the natural in utero history of PVFR-VTI or pulmonary vein size (PVS). In this study, we evaluate trends in fetal PVFR-VTI and pulmonary vein size throughout gestation in HLHS fetuses. **Methods:** We performed a retrospective review of 133 HLHS fetuses evaluated between 17–38 weeks gestation from 2012–2022. Serial PVFR-VTIs and PVS (the largest diameter) were calculated from spectral Doppler and 2D echo. Fetuses with PVFR-VTI < 5 were considered high risk for RAS and EAS occurred within 24 h after birth. We assessed HLHS characteristics, fetal death (voluntary interruption of pregnancy (VIP) vs. intrauterine fetal demise (IUFD)), and postnatal outcomes including EAS, transplant, surgical intervention, and death prior to first operative procedure. Comparisons measures used two-sample t-tests and Fisher’s exact tests. Longitudinal comparisons were completed using linear mixed effects models account for the correlation between repeated measurements. **Results:** HLHS with PVFR-VTI < 5 required EAS more frequently (10/36, 27.8%) than PVFR-VTI > 5 group (3/97, 3.1%, p < 0.001), but 72% with PVFR < 5 had an adequate atrial shunt. Overall, the lowest PVFR-VTI and that obtained latest in gestation was associated with EAS. There were no differences in fetal or neonatal deaths in pregnancies with intent to treat 4.5% and 6%, respectively) based on PVFR-VTI. After EAS, 84% neonates underwent stage 1 palliation and survived. PVFR-VTI > 5 increased by 0.02 per week of gestation (p = 0.02) but did not change during gestation if PVFR < 5 (p = 0.18). PVS increased by 0.004 cm for every week of GA (p < 0.001) and by an average of 0.005 cm for every increase PVFR-VTI by 1 (p = 0.02). **Conclusions:** In fetal HLHS, PVFR-VTI < 5 does not increase during gestation. These trends may be helpful in predicting the need for EAS. Further studies are needed to discriminate the truly restrictive septum needing EAS in fetuses with PVFR-VTI < 5.

## Variation in Prenatal Surveillance and Management of Anti-ro/ssa Autoantibody Positive Pregnancies

Lisa Howley, MD, Stacy Killen, MSCI, MD, Erin Paul, MD, Anita Krishnan, MD, Melanie Gropler, MD, Bailey Drewes, MS, Stephanie Eyerly-Webb, PhD, Eric Dion, BA, Amy Lund, DNP, CPNP, Jill Buyon, MD, Bettina Cuneo, MD.

Lead Centers: Midwest Fetal Care Center at Children’s Minnesota, Minneapolis, MN and Children’s Hospital of Colorado, Aurora, CO.

**Background:** Immune-mediated fetal complete heart block and carditis due to anti-Ro/SSA autoantibodies carries an almost 20% mortality. No universally accepted guidelines exist for clinical management of these patients. We report survey findings on current international prenatal surveillance protocols and treatment strategies for managing anti-Ro/SSA autoantibody positive pregnancies. **Methods:** An electronic REDCap questionnaire was distributed to members of the Fetal Heart Society and the North American Fetal Therapy Network. The survey queried institution-based patient risk stratification, methods and frequency of fetal cardiac surveillance, treatment of varied conduction abnormalities, and post-natal monitoring of anti-Ro/SSA pregnancies. **Results:** 101 participants completed the questionnaire (73% pediatric cardiology and 27% perinatology, 56.5% reporting > 10 years’ experience) at 60 primarily academic centers (59% US, 17% international, 24% no reported center). Most respondents do not risk stratify pregnancies by anti-Ro/SSA titer, and those that do use a variety of titer cutoffs. Respondents considered anti-Ro/SSA status (85%), titer (25%), and/or a prior affected child (79%) when setting monitoring strategies. Echocardiography is the primary tool for fetal surveillance (94%), whereas home handheld Doppler monitoring is used by 28% of respondents and 46% employ weekly or biweekly in-office fetal heart rate monitoring. The frequency of monitoring varied widely for all surveillance methods. Respondents reported treating 1° (40%), 2° (78%), and 3° (67%) fetal heart block primarily with dexamethasone ± IVIG. Post-natal ECG and echocardiograms are completed in 77% and 37% of newborns, respectively. Only 12% of all respondents refer to literature guidelines for their practice decisions, while 83% rely on experience. **Conclusion:** Our survey results confirmed that wide practice variation, primarily experiential, exists in current fetal cardiac surveillance and treatment approaches for anti-Ro/SSA autoantibody positive pregnancies, highlighting the need for evidence-based protocols.

## Postnatal Outcomes of Prenatally Diagnosed Double Outlet RIGHT Ventricle

Haligheri Geetha, MD^1^, Kathol Melanie, RDMS^1^, France Rita, RDMS^1^, Komarlu Rukmini, MD ^2^, Parthiban Anitha, MD^3^.

^1^Children’s Mercy Hospital, Kansas; ^2^Cleveland Clinic Foundation, Cleveland, OH; ^3^Texas Children’s Hospital, TX.

**Background:** Double Outlet Right Ventricle (DORV) is a rare complex congenital heart defect accounting for < 1% of congenital heart disease (CHD). We sought to assess the spectrum of postnatal presentation of prenatally diagnosed DORV and associated anomalies. **Methods:** This was a single center retrospective review of fetal echocardiograms from 2000 to 2020. All fetuses with fetal diagnosis of DORV were included. Fetuses without echocardiograms for review and those whose postnatal status was unknown were excluded. Maternal data including associated comorbid conditions, gestational age at diagnosis and prenatal genetic testing. Fetal data included type of DORV, presence of AV valve regurgitation, associated cardiac anomalies, presence of IUGR, arrhythmias, need for prostaglandin and anticipated repair. Postnatal data included anatomic diagnosis, associated cardiac anomalies, extracardiac and genetic diagnoses as well as immediate postnatal outcomes. **Results:** 42/1252 fetuses had prenatal diagnosis of DORV. The mean gestational age at diagnosis was 25.4 weeks (19-36 weeks), mean maternal age was 27.5 years (17-39 years). Maternal comorbidities included diabetes (0.09%), hypertension (0.07%) and AMA (0.09%). 18 fetuses had genetic testing, with 4/18 (22%) having chromosomal anomalies. 18/42 (42%) had multiple anomalies. 14/42 (43%) patients were counseled as potential biventricular (BV) repair, 22/42 (57%) were counseled as potential single ventricle (SV) palliation: 11/22 (50%) had hypoplastic left heart variant; 2/22 (0.09%) had pulmonary atresia and hypoplastic right heart and 9/22 (40.9%) had unbalanced atrioventricular septal defects with Heterotaxy (2 patients had straddling AV valves and VSD anatomy unsuitable for septation). 6/42 ((14.2%) fetuses were deemed to potentially be a biventricular or single ventricular palliation. All fetuses were live born. 6/42 (14.2%) of fetuses died postnatally; 4 in the SV group; 1 in the BV group. 1 died as comfort care was chosen. 7/42(16.6%) were premature. Anatomic diagnosis was accurate in 86% and predictive accuracy of need for prostaglandin infusion was 91%. Accuracy of prediction for BV repair was 85.7%. Accuracy of prediction for SV repair was 95% with 6/24 (25%) fetuses having potential for future BV repair. **Conclusions:** DORV is a heterogeneous CHD with variable physiology. The presence of chromosomal anomalies and other congenital anomalies was a poor prognostic factor. BV subtype was associated with better outcomes. Prenatal prediction of appropriate palliation is fairly accurate.

## The Yield of Postnatal Screening for Extracardiac Anomalies in Neonates with Prenatally Diagnosed Critical Congenital Heart Disease

Authors: Deani H. McVadon MD (1), Rachel A. Wyand MD (1), Teresa Kontos MD (1), Rachel Kaye MD (1), Anthony M. Hlavacek MD (1), Geoffrey A. Forbus MD (1), Sinai C. Zyblewski MD (1), Taufiek K. Rajab MD (1), Andrew M. Atz MD (1), Leslie Spence MD (1), John M. Costello MD MPH (1).

Affiliations: (1) Medical University of South Carolina, Charleston, SC USA.

**Background:** Many centers utilize a universal postnatal screening protocol for non-cardiac anomalies and genetic syndromes in neonates born with critical congenital heart disease (CCHD). With improvements in fetal imaging and prenatal genetic testing, many extra-cardiac anomalies and syndromes are diagnosed prenatally. We sought to determine the yield of *postnatal* screening for major non-cardiac structural anomalies and genetic syndromes in newborns prenatally diagnosed with CCHD who were not thought on prenatal testing or initial postnatal exam to have associated anomalies. We hypothesized that the yield of such postnatal screening would be relatively low. **Materials and Methods:** This single center retrospective cohort study included all neonates with a prenatal diagnosis of CCHD who underwent neonatal cardiac surgery at our center between 2012 and 2021. Maternal records were reviewed for fetal testing including cardiac and non-cardiac imaging, genetic testing, and confirmation or concern for extra-cardiac anomalies and/or syndromes. In patients without any prenatal concern for non-cardiac issues, postnatal testing was reviewed including echocardiogram, renal ultrasound, cranial ultrasound, and genetic testing. **Results:** Of the 502 neonates with CCHD who underwent cardiac surgery during the study period, 304 were prenatally diagnosed with CCHD (60.6%). Of these, 157 had no concern prenatally for extra-cardiac abnormalities or genetic syndromes and no concerning postnatal physical exam findings. The yield of screening postnatal cranial and renal ultrasound screening for important extra-cardiac anomalies in this group were both relatively low (2%), while the yield of genetic testing in this group was higher (10%). **Conclusion:** While the yield of postnatal ultrasound screening for extra-cardiac anomalies was low, it still may be considered worthwhile as the identified anomalies had acute management and/or prognostic implications. The higher yield of postnatal genetic testing provides additional support for universal postnatal genetic screening in neonates with CCHD and is reflective of the limitations of non-invasive prenatal genetic testing prenatally.

## Transposition of the Great Arteries with Intact Ventricular Septum: 12-Year Experience and Perinatal Outcomes of Early Surgical Correction Using Autologous Umbilical Cord Blood

Kurkevych Andrii, MD, PhD^1^, Yemets Illya, MD, PhD.^2^

^1^Research and Diagnosis Department, ^2^ Department of Cardiac Surgery of Newborns and Early Childhood, Ukrainian Children’s Cardiac Center, Kyiv, Ukraine.

**Background:** Arterial switch operation (ASO) is the surgery of choice in neonates with transposition of the great arteries with intact ventricular septum (TGA-IVS). In this study, we analyzed our 12-year experience performing ASO in the first hours of life using autologous umbilical cord blood (AUCB) transfusion (the CorD program) in newborns with prenatally diagnosed TGA-IVS. **Materials and methods:** Between 2009 and 2020, 271 fetuses were prenatally diagnosed with TGA-IVS at our institution (UCCC). According to the CorD program, all fetuses were born near UCCC in Kyiv. During delivery, AUCB was collected and all newborns were urgently transported to UCCC. **Results:** The mean age of 271 fetuses at the initial diagnosis of TGA-IVS was 26,8 ± 6,2 weeks. Three (1,1%) pregnancies were terminated. In 15 (5,5%) cases, follow-up and perinatal results were lost. Of the remaining 253 cases, there were 10 (4,0%) early preoperative deaths, mainly due to premature birth. In 164 (64,8%) cases (CorD group), ASO was performed in the first hours of life without preoperative balloon atrial septostomy (BAS). In 89,0% (n = 146) cases, it was possible to use AUCB, while in 11,0% of cases there were contraindications. There were 3 (1,8%) early postoperative deaths. In the other 79 (31,2%) cases (non-CorD group) ASO was performed after 2 days of life (mean age 5,1 + 2,9 days). The main reason was the need to perform urgent BAS procedure (74 cases, or 93,7%) due to restrictive foramen ovale, premature birth and twin pregnancy. Isolated AUCB was used only in 25,3%, and together with donor blood in another 20,3%. There were 3 (3,8%) early postoperative deaths. **Conclusions:** Prenatal diagnosis of TGA-IVS allowed in most cases to successfully operate on newborns in the first hours of life without BAS and donor blood. Restrictive foramen ovale, premature birth and twin pregnancy remained the main problems in this group.

## Best Abstract Award

### Patient-Reported Barriers to Prenatal Diagnosis of Congenital Heart Defects: A Prospective, Mixed-Methods Study

**Authors:** Sivakumar, Adithya, BE;^1^ Burton, Shelvonne;^2^ Gandhi, Rupali, MD, JD;^3^ Yee, Lynn, MD, MPH;^4^ Johnson, Joyce, MD, MSc;^5^ Patel, Angira, MD, MPH;^2^ Woo, Joyce, MD, MS.^2^

^1^Rush University, Chicago, IL. ^2^Ann & Robert H. Lurie Children’s Hospital, Northwestern University Feinberg School of Medicine, Chicago, IL. ^3^Advocate Children’s Hospital, Chicago, IL. ^4^Northwestern University Feinberg School of Medicine, Chicago, IL. ^5^Johns Hopkins All Children’s Hospital, Baltimore, MD. Research performed at Ann & Robert H. Lurie Children’s Hospital, Northwestern University Feinberg School of Medicine, Chicago, IL.

**Background/Hypothesis:** The sensitivity of fetal echocardiography in the prenatal diagnosis of congenital heart defects (CHDs) approaches 90%, yet national prenatal diagnosis rates of CHD are only 30–50%. Previous analyses with large administrative data have revealed socioeconomic factors, such as public insurance, to be associated with disparate prenatal diagnosis rates. More granular, prospective data can identify modifiable barriers to prenatal CHD diagnosis. This study elucidates patient-reported barriers to prenatal CHD diagnosis within the Chicagoland area. **Materials and Methods:** Semi-structured telephone surveys were administered to mothers of children who had CHD surgery at Lurie Children’s Hospital between 2019–2020. The survey was developed using a mixed-methods, convergent parallel design with closed and open-ended questions about perceived barriers to prenatal CHD diagnosis. Quantitative data were summarized with descriptive statistics, while qualitative data were coded to thematic domains. **Results:** In total, 39 respondents completed the survey. Of these, 26% (n = 10) were of Hispanic ethnicity, 5% (n = 2) were of non-Hispanic Black race, 67% (n = 26) received fetal echocardiogram, and 64% (n = 25) reported a barrier to prenatal diagnosis. Reported barriers were matched into three themes (Fig. 1): perceived inefficiency with prenatal care (n = 7, 18%), difficulty obtaining access to a fetal echocardiogram (n = 5, 13%), and no fetal echocardiogram obtained (n = 13, 33%). Within these themes, responses included missed diagnoses by obstetric ultrasound or fetal echocardiogram (n = 12), lack of prenatal or maternal–fetal medicine care (n = 2), difficulty obtaining insurance approval (n = 2), and distance to care (n = 2). Of the reported barriers, only two were specifically related to the COVID-19 pandemic. **Conclusions:** Barriers to prenatal diagnosis of CHD are not limited to missed diagnoses by prenatal imaging. Public health barriers such as difficulty accessing obstetric or maternal–fetal-medicine care, geographic barriers such as distance to care, and systems-level barriers such as insurance approval, are modifiable targets for intervention by institutional, local, and regional healthcare policy.

**Fig. 1:**
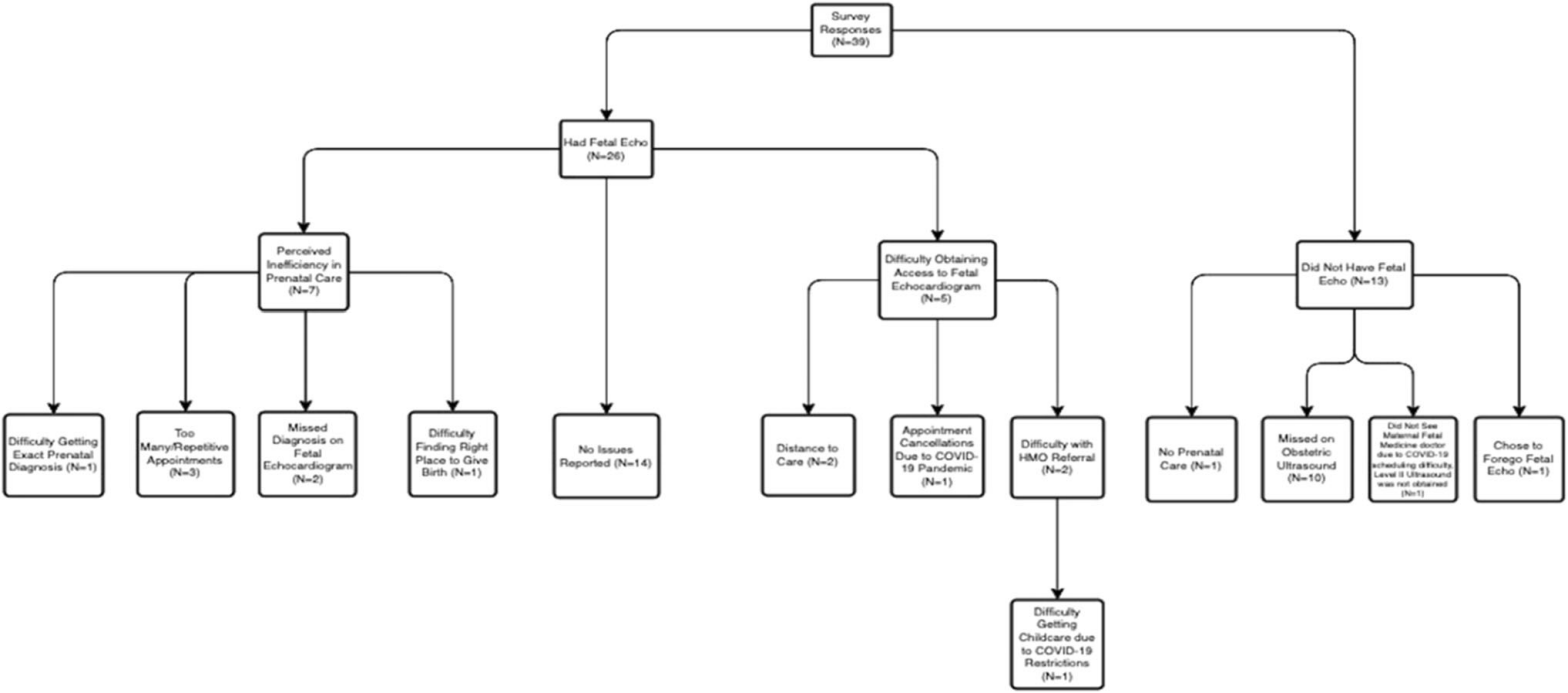
Categorization of barriers reported among mothers of children diagnosed with congenital heart disease

**Table 1:** HLHS characteristics and outcomes by FR-VTI subgroups

Covariate	Overall	Forward/Reverse VTI < 5	Forward/Reverse VTI > 5	p-value
	(N = 133)	(N = 36)	(N = 97)	
GA at Delivery (weeks)	38.0 (2.4)	37.9 (2.8)	38.0 (2.3)	0.944
Birthweight (kg)	3.1 (0.6)	3.1 (0.7)	3.0 (0.6)	0.539
Diagnosis				0.551
MA/AA	49 (36.8%)	14 (38.9%)	35 (36.1%)	
MA/AS	3 (2.3%)	0 (0.0%)	3 (3.1%)	
MS/AA	48 (36.1%)	14 (38.9%)	34 (35.1%)	
MS/AS	33 (24.8%)	8 (22.2%)	25 (25.8%)	
PVFR-VTI				
GA at Last FR-VTI	34.2 (5.0)	33.9 (5.3)	34.2 (4.9)	0.763
Last FR-VTI	11.6 (10.1)	4.1 (2.4)	14.6 (10.5)	< 0.001
GA at Lowest FR-VTI	29.3 (6.3)	31.0 (5.9)	28.7 (6.3)	0.046
Lowest FR-VTI	8.7 (7.4)	3.3 (1.3)	10.8 (7.8)	< 0.001
Outcomes				
Fetal Demise	17 (14.7%)	7 (19.4%)	12 (12.4%)	0.402
VIP	11 (8.3%)	3 (8.3%)	8 (8.3%)	1.0
IUFD	6 (4.5%)	3 (8.3%)	3 (3.1%)	0.344
Septostomy < 24 Hours	13 (9.8%)	10 (27.8%)	3 (3.1%)	< 0.001
Norwood	98 (73.7%)	27 (75%)	71 (73.2%)	
Hybrid	3 (2.3%)	0 (0.0%)	3 (3.6%)	
Transplant	3 (2.3%)	0 (0.0%)	3 (3.6%)	
Death prior to first operative procedure	8 (6%)	2 (5.6%)	7 (7.2%)	

*IUFD* intrauterine fetal demise, *VIP* voluntary interruption of pregnancy, *PVFR-VTI* pulmonary vein forward/reverse velocity time integral

